# Draft Genome Assemblies of *Proteus mirabilis* ATCC 7002 and *Proteus vulgaris* ATCC 49132

**DOI:** 10.1128/genomeA.01064-14

**Published:** 2014-10-23

**Authors:** T. D. Minogue, H. E. Daligault, K. W. Davenport, K. A. Bishop-Lilly, D. C. Bruce, P. S. Chain, S. R. Coyne, O. Chertkov, T. Freitas, K. G. Frey, J. Jaissle, G. I. Koroleva, J. T. Ladner, G. F. Palacios, C. L. Redden, Y. Xu, S. L. Johnson

**Affiliations:** a Diagnostic Systems Division (DSD), United States Army Medical Research of Infectious Disease (USAMRIID), Fort Detrick, Maryland, USA; b Los Alamos National Laboratory (LANL), Los Alamos, New Mexico, USA; c Naval Medical Research Center (NMRC)-Frederick, Fort Detrick, Maryland, USA; d Henry M. Jackson Foundation, Bethesda, Maryland, USA; e Center for Genome Sciences (CGS), USAMRIID, Fort Detrick, Maryland, USA

## Abstract

The pleomorphic swarming bacilli of the genus *Proteus* are common human gut commensal organisms but also the causative agents of recurrent urinary tract infections and bacteremia. We sequenced and assembled the 3.99-Mbp genome of *Proteus mirabilis* ATCC 7002 (accession no. JOVJ00000000) and the 3.97-Mbp genome of *Proteus vulgaris* ATCC 49132 (accession no. JPIX00000000), both of which are commonly used reference strains.

## GENOME ANNOUNCEMENT

As members of the *Enterobacteriaceae* (Gram-negative bacilli), bacteria of the genus *Proteus* are commonly found in the human intestinal tract and are associated with opportunistic infections (1). The genus consists of four species (*Proteus mirabilis*, *Proteus myxofaciens*, *Proteus penneri*, and *Proteus vulgaris*), three of which cause human disease (*P. myxofaciens* is an insect pathogen) (1). Members of the genus (primarily *P. mirabilis* but also *P. penneri* and *P. vulgaris*) are the third most common causes of complicated urinary tract infections in the United States (1, 2) and are notable for their swarming abilities, which are directly linked to their ability to initiate disease (2, 3). Here, we sequenced two isolates, both originally isolated from clinical samples and both commonly used in quality control testing: *P. mirabilis* ATCC 7002 and *P. vulgaris* ATCC 49132.

High-quality genomic DNA was extracted from purified isolates of each strain using the Qiagen Genomic-tip 500 at the U.S. Army Medical Research Institute of Infectious Diseases-Diagnostic Systems Division (USAMRIID-DSD). Specifically, 100-ml bacterial cultures were grown to stationary phase and nucleic acid extracted as per the manufacturer’s recommendations. The draft genomes of *P. mirabilis* ATCC 7002 and *P. vulgaris* ATCC 49132 included a combination of Illumina (4) and 454 technologies (5). For each genome, we constructed and sequenced a 100-bp Illumina library (*P. mirabilis*, 307-fold genome coverage; *P. vulgaris*, 300-fold genome coverage) and a long-insert paired-end 454 library (*P. mirabilis*, 8,695.7 ± 2,174-bp insert and 24-fold genome coverage; *P. vulgaris*, 8,041 ± 2,010-bp insert and 28-fold genome coverage). The 454 paired-end data were assembled in Newbler (6), and those consensus sequences were computationally shredded into 2-kbp overlapping fake reads (shreds). The Illumina sequencing data were assembled with Velvet (6), and the consensus sequences were computationally shredded into 1.5-kbp overlapping shreds. All data were additionally assembled in AllPaths (7), and the consensus sequences were computationally shredded into 5-kbp overlapping shreds. We integrated the 454 Newbler consensus shreds, Illumina Velvet consensus shreds, AllPaths consensus shreds, and the 454 paired-end library read pairs using parallel Phrap version (High Performance Software, LLC). Possible misassemblies were corrected using in-house scripts and manual editing in Consed (8–10).

Automatic annotation for each genome utilized an Ergatis-based (11) workflow at the Los Alamos National Laboratory (LANL), with minor manual curation. The *P. mirabilis* ATCC 7002 genome (accession no. JOVJ00000000) is 3,992,612 bp (38.8% G+C content) and contains 3,631 coding sequences (CDSs), 11 rRNAs, and 77 tRNAs in 15 contigs. The *P. vulgaris* ATCC 49132 genome (accession no. JPIX00000000) is 3,972,483 bp (37.9% G+C content) and contains 3,592 CDSs, 12 rRNAs, and 77 tRNA in 12 contigs. Each strain contains at least nine of the genes putatively implicated in swarming (2).

### Nucleotide sequence accession numbers.

The NCBI accession no. for *P. mirabilis* ATCC 7002 is JOVJ00000000, and for *P. vulgaris* ATCC 49132, it is JPIX00000000.
